# FROM GEROSCIENCE TO GEROTHERAPEUTICS: KUAKINI CENTER OF RESEARCH EXCELLENCE FOR TRANSLATIONAL RESEARCH ON AGING

**DOI:** 10.1093/geroni/igae098.3660

**Published:** 2024-12-31

**Authors:** Bradley Willcox, Kazuma Nakagawa, Yeonjung Lee, Kamal Masaki, Richard Allsopp

**Affiliations:** Kuakini Medical Center, Honolulu/Kuakini Medical Center, Hawaii, United States; Kuakini Medical Center, Honolulu, Hawaii, United States; University of Hawaiʻi at Mānoa/Kuakini Medical Center, Honolulu, Hawaii, United States; Kuakini Medical Center, Honolulu/Kuakini Medical Center, Hawaii, United States; University of Hawaii, Honolulu, Hawaii, United States

## Abstract

The primary aim of the Kuakini HHP Center of Excellence for Translational Research on Aging is to utilize geroscientific principles to improve human healthspan, including pharmacology-based gerotherapeutics. This research leverages an exceptional cohort of 8,006 American men of Japanese and Okinawan ancestry, aged 45-68 years at the baseline exam in 1965. Fourteen comprehensive clinical exams have been conducted. Over 500,000 biological samples and over 900 brains have been collected over six decades. The primary aim of our Center is to translate these largely NIH-funded resources into therapeutic innovations that modify aging’s biological pathways, potentially preventing or alleviating multiple age-related diseases and optimizing human healthspan. Our early-stage investigators have novel, recent data on risk factors for premature cognitive aging. Our research approach, with our biotech collaborators, has also contributed to a data science ecosystem with AI and ML-enabled insights on healthy aging from novel multi-omic studies and aging-related health outcomes from our human aging cohort. Key findings are consistent with the geroscience hypothesis and have identified specific pathways for targeted gerotherapeutic interventions, now in Phase 2 human trials, that may contribute to healthier human aging. This presentation will present the latest findings regarding the specific pathways, the molecular targets, and the gerotherapeutic agents that hold promise to mitigate major aging-related diseases, including sarcopenia and cognitive impairment.

